# A multi-trait meta-analysis with imputed sequence variants reveals twelve QTL for mammary gland morphology in Fleckvieh cattle

**DOI:** 10.1186/s12711-016-0190-4

**Published:** 2016-02-16

**Authors:** Hubert Pausch, Reiner Emmerling, Hermann Schwarzenbacher, Ruedi Fries

**Affiliations:** Lehrstuhl fuer Tierzucht, Technische Universitaet Muenchen, 85354 Freising, Germany; Institut fuer Tierzucht, Bayerische Landesanstalt fuer Landwirtschaft, 85586 Poing, Germany; ZuchtData EDV Dienstleistungen GmbH, 1200 Vienna, Austria

## Abstract

**Background:**

The availability of whole-genome sequence data from key ancestors in bovine populations provides an exhaustive catalogue of polymorphic sites that segregate within and across cattle breeds. Sequence variants identified from the sequenced genome of key ancestors can be imputed into animals that have been genotyped using medium- and high-density genotyping arrays. Association analysis with imputed sequences, particularly when applied to multiple traits simultaneously, is a very powerful approach to detect candidate causal variants that underlie complex phenotypes.

**Results:**

We used whole-genome sequence data from 157 key ancestors of the German Fleckvieh cattle population to impute 20,561,798 sequence variants into 10,363 animals that had (partly imputed) genotypes based on 634,109 single nucleotide polymorphisms (SNPs). Rare variants were more frequent among the sequence-derived than the array-derived genotypes. Association studies with imputed sequence variants were performed using seven correlated udder conformation traits as response variables. The calculation of an approximate multi-trait test statistic enabled us to detect 12 quantitative trait loci (QTL) (P < 2.97 × 10^−9^) that affect different morphological features of the mammary gland. Among the tested variants, the most significant associations were found for imputed sequence variants at 11 QTL, whereas the top association signal was observed for an array-derived variant at a QTL on bovine chromosome 14. Seven QTL were associated with multiple phenotypes. Most QTL were located in non-coding regions of the genome but in close proximity of candidate genes that could be involved in mammary gland morphology (*SP5*, *GC*, *NPFFR2*, *CRIM1*, *RXFP2*, *TBX5*, *RBM19* and *ADAM12*).

**Conclusions:**

Using imputed sequence variants in association analyses allows the detection of QTL at maximum resolution. Multi-trait approaches can reveal QTL that are not detected in single-trait association studies. Most QTL for udder conformation traits were located in non-coding regions of the genome, which suggests that mutations in regulatory sequences are the major determinants of variation in mammary gland morphology in cattle.

**Electronic supplementary material:**

The online version of this article (doi:10.1186/s12711-016-0190-4) contains supplementary material, which is available to authorized users.

## Background

Genome-wide association studies (GWAS) using dense single nucleotide polymorphisms (SNPs) have facilitated the identification of quantitative trait loci (QTL) for numerous phenotypes. However, because of the extensive linkage disequilibrium (LD) that is present in bovine populations, the QTL regions are often very large, which makes the identification of underlying genes and variants usually inefficient. Moreover, the current genotyping arrays interrogate only a limited number of polymorphic sites that are primarily located in non-coding regions of the genome [1].

The availability of whole-genome sequences has led to an exhaustive catalogue of polymorphic sites that segregate within and across bovine populations [2, 3]. However, the costs of sequencing the whole genome for a large number of animals are still high, thus, using a relatively low number of sequenced key ancestors as reference animals to impute sequence variants into any animal for which dense genotyping data are available, is an interesting alternative [4, 5]. Association studies with imputed sequence variants can then pinpoint candidate causal variants that control complex trait variation [3, 6].

Computationally efficient algorithms have been developed to perform association studies on thousands of individuals that are genotyped at millions of polymorphic sites (e.g., [7, 8]). In cattle, such association studies are generally performed within breeds on a trait-by-trait basis and by testing one variant at a time. Association analyses that include multiple phenotypes may be more powerful to identify QTL for complex traits, particularly in the case of causal variants that affect multiple correlated phenotypes [9]. However, a multivariate test of association for millions of sequence variants and many phenotypes is computationally challenging [9]. An approximate multi-trait test statistic allows to efficiently combine the results of multiple association studies that were performed separately and thereby increases the power to identify trait-associated variants [10].

Udder conformation traits are routinely recorded in bovine populations during the evaluation of first-crop daughters of test bulls. Phenotypes for such traits are an important source of information because mammary gland morphology is highly correlated with mastitis susceptibility and productive life span [11–13]. Although the definitions of udder conformation traits may vary across breeds, phenotypic records generally describe the morphology and placement of teats and the overall shape of the mammary gland. The heritability of such traits is relatively high and ranges from 0.14 to 0.42 and most traits related to mammary gland morphology are correlated with each other [11, 14].

In this paper, we present results that are based on the imputation of whole-genome sequence variants into more than 10,000 Fleckvieh animals that were genotyped using dense SNP arrays. We performed association studies with more than 16 million sequence variants using seven highly correlated udder conformation traits as response variables. The multi-trait meta-analysis enabled us to detect 12 QTL that affect different morphological features of the mammary gland in Fleckvieh cattle.

## Methods

### Animal ethics statement

DNA of artificial insemination bulls was prepared from semen samples that were collected by approved commercial artificial insemination stations as part of their regular breeding and reproductive measures in cattle industry. No ethical approval was required for this study.

### Genotypes of the target population

All animals were genotyped using medium- and high-density SNP arrays. The high-density dataset consisted of 3545 Fleckvieh animals that had previously been genotyped with the Illumina BovineHD Bead chip that includes 777,962 SNPs. The medium-density dataset consisted of 7073 Fleckvieh animals that had been genotyped with the Illumina BovineSNP50 Bead chip (version 1 and version 2) comprising approximately 54,000 SNPs. Chromosomal positions of SNPs were based on the UMD3.1 assembly of the bovine genome [15]. Mitochondrial, X-chromosomal, Y-chromosomal SNPs and SNPs that had no known chromosomal position were not considered for further analyses. Quality controls were carried out separately for each dataset as described by Pausch et al. [16] and briefly indicated here: SNPs were retained if they had a call rate per SNP and a call rate per individual greater than 90 % and a minor allele frequency (MAF) higher than 0.5 % and if they showed no deviation from the Hardy–Weinberg equilibrium (P > 0.0001) and no pedigree conflicts. After quality control, the medium-density genotypes were imputed to higher density using a combination of *Beagle* [17] and *Minimac* [18] as described by Pausch et al. [5]. Only SNPs with a MAF higher than 0.5 % were retained after imputation. The final array-derived dataset consisted of 10,363 animals with (partly imputed) genotypes at 634,109 autosomal SNPs.

### Generation of sequence data

We used whole-genome sequence data from 263 animals that represented 10 bovine breeds, among which the Fleckvieh breed with 157 animals. Most of the sequenced animals were key ancestors for their breeds [19]. The generation and analysis of whole-genome sequence data are described by Pausch et al. [20]. SNPs, short insertions and deletion polymorphisms were genotyped for all 263 sequenced animals simultaneously using the multi-sample approach implemented in the *mpileup* module of *SAMtools* [21] and in a variant calling pipeline described by Jansen et al. [2]. A total of 25,426,490 polymorphic sites were identified. The functional effects of the sequence variants were analyzed using the annotation of the UMD3.1 assembly of the bovine genome [22] and as described by Jansen et al. [2].

### Imputation of sequence variants

The imputation reference panel consisted of 157 sequenced Fleckvieh animals with 20,561,798 autosomal variants. Haplotypes were inferred using *Beagle* [17] and served as reference to impute genotypes for 20,561,798 variants into 10,363 target animals with (partly imputed) genotypes at 634,109 SNPs (see above) using *Minimac* [18].

### Phenotypes tested for association

Response variables for the association tests were daughter yield deviations (DYD) with an accuracy greater than 0.7 for seven udder conformation traits (teat thickness, teat length, teat position, udder depth, central ligament, fore udder attachment and fore udder length). Depending on the trait, the number of animals with phenotypes ranged from 5470 to 7159 (Table 1). In addition, association tests with 682,047 imputed sequence variants located on BTA14 (BTA for *Bos taurus* chromosome) were carried out on 6838 animals using DYD for height at the sacral bone as response variable.

**Table 1:** Characteristics of seven udder conformation traits

Phenotype (and abbreviation)	Number of animals with phenotypes	Distribution of the DYD			Accuracy of the DYD		
		Min	Max	Mean (±sd)	Min	Max	Mean (±sd)
Udder depth (UD)	7110	−2.22	2.75	0.11 (±0.64)	0.71	0.99	0.91 (±0.04)
Teat thickness (TT)	7108	−2.32	2.17	0.04 (±0.52)	0.71	0.99	0.91 (±0.04)
Teat length (TL)	7159	−2.61	2.52	−0.02 (±0.66)	0.71	0.99	0.93 (±0.04)
Teat placement (TP)	7108	−1.92	2.50	0.16 (±0.55)	0.71	0.99	0.91 (±0.04)
Fore udder length (FUL)	7034	−2.03	2.15	0.21 (±0.51)	0.71	0.99	0.88 (±0.04)
Central ligament (CL)	6923	−2.24	2.32	0.11 (±0.57)	0.71	0.99	0.85 (±0.05)
Fore udder attachment (FUA)	5470	−2.73	2.48	0.13 (±0.65)	0.71	0.99	0.87 (±0.05)

### Single-trait genome-wide association studies

We considered 16,816,809 imputed sequence variants with a MAF higher than 0.5 % for the GWAS. The imputed sequence variants were tested for association with each trait in turn using a two-step variance component-based approach that accounts for population stratification and the resulting inflation of false positive association signals by fitting a genomic relationship matrix. The *EMMAX* software tool [7] was used to fit the model **y** = μ + **u** + **e**, where y is a vector of DYD, µ is the intercept, **u** is a vector of additive genetic effects ~ N(0,${\, \mathbf{G}}{{\upsigma }}_{\text{a}}^{2}$), where **G** is the realized genomic relationship matrix that was built based on the genotypes of 634,109 autosomal SNPs for the 10,363 animals following VanRaden’s approach [23] and σ_a_^2^ is the additive genetic variance. **e** is a vector of residuals ~ N(0,${\, \mathbf{I}}{{\upsigma }}_{\text{e}}^{2}$), where **I** is the identity matrix and σ_*e*_^2^ is the error variance. In a second step, the allele substitution effect (b) was obtained from a generalized linear regression model: **y** = μ + **x**b + **η**, where **x** is a vector of expected allele dosages and **η** is a vector of random residual deviates with variance **G**σ_a_^2^ + **I**σ_e_^2^. Inflation factors were calculated using the *estlamdba()*-function of *GenABEL* [24]. Sequence variants with a P value less than 2.97 × 10^−9^ were considered as significantly associated (5 % Bonferroni-corrected significance threshold for 16,816,809 independent tests). The proportion of DYD variance explained per QTL was estimated by $\frac{{2{\text{p}}\left( {1 - {\text{p}}} \right){{\upalpha }}^{2} }}{{{{\upsigma }}^{2} }}$, where p and *α* are the frequency and substitution effect, respectively, of the minor QTL allele and *σ*^2^ is the DYD variance. An analysis conditional on the most significantly associated variant was carried out by taking the expected allele dosages of the top variant as covariates in the linear regression model (see above).

### Multi-trait meta-analysis

An approximate multi-trait test statistic was calculated for 16,816,809 imputed sequence variants using ${{\upchi }}_{{{\text{df}} \, = \, {\text{n}}}}^{2} = {\mathbf{t}}_{{\mathbf{i}}}^{'} {\mathbf{V}}^{ - 1} {\mathbf{t}}_{{\mathbf{i}}}$, where n is the number of traits, **t**_**i**_ is a n × 1 vector of t values (${\text{t}} = \frac{\text{b}}{{{\text{se}}({\text{b}})}}$) of the ith SNP and **V**^**−1**^ is the inverse of the n × n correlation matrix of t values [10]. The correlation matrix **V** was constructed from t values of 16,816,809 imputed sequence variants. Sequence variants with a P_META_ value less than 2.97 × 10^−9^ were considered as significantly associated (see above).

## Results

**Fig. 1:**
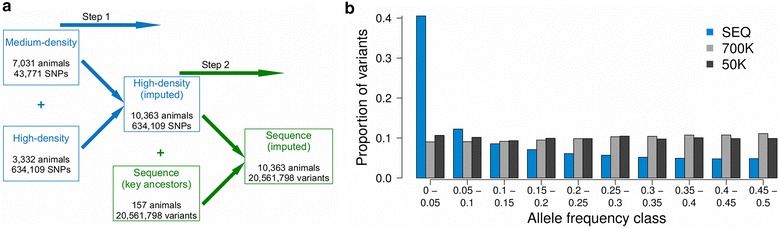
Imputation of sequence variants. **a** Overview of the stepwise imputation of 20,561,798 sequence variants into 10,363 Fleckvieh animals. **b** Distribution of allele frequencies of imputed and array-derived variants. *Blue* and shades of *grey* represent the proportion of imputed sequence (SEQ) and array-derived (50 K, 700 K) variants, respectively, for ten allele frequency classes

More than 20 million sequence variants were imputed into 10,363 animals that had (partly imputed) array-derived genotypes for 634,109 SNPs (Fig. 1a). The distributions of the allele frequencies of the imputed sequence (SEQ) variants and of the array-derived variants differed. Variants from medium- (50 K) and high-density (700 K) SNP arrays were almost uniformly distributed across different MAF classes, whereas the imputed sequence variants were enriched for low-frequency MAF classes (Fig. 1b). The proportions of variants with a MAF lower than 0.05 were equal to 10.66, 9.04 and 40.52 % and the average MAF was equal to 0.249, 0.260 and 0.145 for the 50 K, 700 K and SEQ datasets, respectively.

### Association studies with udder conformation traits

**Fig. 2:**
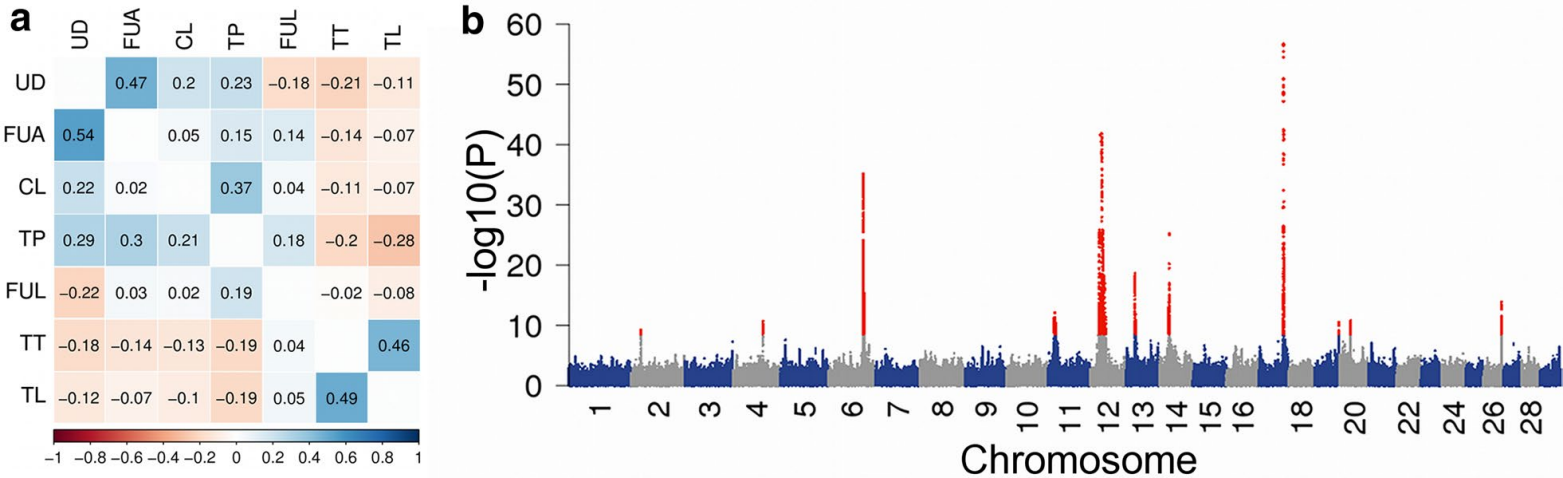
Identification of 12 QTL for mammary gland morphology. **a** Correlations between seven udder conformation traits. The values in the upper and lower triangles represent genomic correlations calculated with the signed t values and genetic correlations, respectively. The abbreviations of the traits are in Table 1. Shades of *blue* and *red* indicate positive and negative correlation coefficients, respectively. Genetic correlations were provided from the Institute of Animal Breeding from Bavarian State Research center for Agriculture. **b** Manhattan plot representing the association of 16,816,809 imputed sequence variants in the multi-trait meta-analysis. *Red* *color* represents variants with a P_META_ less than 2.97 × 10^−9^

We performed association studies between 16,816,809 imputed sequence variants with a MAF higher than 0.5 % and DYD for seven udder conformation traits (Table 1). The inflation factors of the association studies ranged from 1.04 (central ligament) to 1.08 (udder depth) with an average inflation factor of 1.06, which indicated that population stratification was under control. The number of QTL detected per trait ranged from zero (fore udder attachment) to five (fore udder length and teat placement) [See Additional file 1: Figure S1]. Correlation coefficients between the seven traits were calculated with the signed t values (i.e., allele substitution effect divided by its standard error, Fig. 2a). The highest correlations were observed between udder depth and fore udder attachment (r = 0.47), teat length and teat thickness (r = 0.46) and central ligament and teat placement (r = 0.37).

A meta-analysis of the seven single-trait association studies revealed 11 QTL (P_META_ <2.97 × 10^−9^) located on 11 chromosomes (Fig. 2b; Table 2). The most significantly associated variants were imputed sequence variants at ten QTL and the top association signal was found for an array-derived SNP at the QTL on BTA14. The multi-trait meta-analysis revealed a QTL on BTA2 (P_META_ = 6.1 × 10^−10^) that was not detected in the single-trait association studies. Closer inspection revealed that the QTL on BTA2 was associated with fore udder attachment (P_SINGLE_ = 1.13 × 10^−6^), teat thickness (P_SINGLE_ = 2.68 × 10^−4^), fore udder length (P_SINGLE_ = 4.37x10^−3^) and udder depth (P_SINGLE_ = 5.01 × 10^−3^), although the P_SINGLE_ values were above the Bonferroni-corrected genome-wide significance threshold. Four QTL on BTA14, 21, 27 and 29 that were associated with teat placement, udder depth, teat length and udder depth, respectively, were not identified in the multi-trait meta-analysis [See Additional file 1: Figure S1]. Closer inspection of these four regions in the multi-trait meta-analysis showed an association (7.91×10^−8^ < P_META_ < 1.98 × 10^−6^), but not at the genome-wide significance.

**Table 2:** Chromosomal positions of 12 QTL for udder morphology detected in the meta-analysis

Chr	Position	NCBI reference ID	MAF	P_META_	Candidate gene(s)
2	25 887 326	rs718110021	0.25	6.1 × 10^−10^	*SP5*
4	75 817 266	rs474527669	0.24	1.9 × 10^−11^	–
6	88 723 742	rs436532576	0.47	7.4 × 10^−36^	*GC, NPFFR2*
6^a^	90 366 765	rs516920231	0.21	8.5 × 10^−16^	*RASSF6*
11	18 757 907	rs384497140	0.28	7.5 × 10^−13^	*CRIM1*
12	29 248 113	rs381918313	0.03	1.6 × 10^−42^	*RXFP2*
13	22 699 039	rs109467286	0.29	2.2 × 10^−19^	–
14	25 015 640	rs109815800	0.12	6.2 × 10^−26^	*PLAG1*
17	62 694 032	rs109134926	0.25	2.2 × 10^−57^	*TBX5, RBM19*
19	61 493 292	rs379185255	0.35	3.4 × 10^−11^	–
20	27 355 591	rs378730330	0.15	1.8 × 10^−11^	–
26	46 058 305	rs42442597	0.34	1.3 × 10^−14^	*ADAM12*

The positions of the QTL correspond to the UMD3.1 assembly of the bovine genome

*Chr* chromosome number, *MAF* minor allele frequency

^a^Marks a QTL that was detected in the conditional analysis

To test if a QTL was completely tagged by the top variant, the most significantly associated variant was fitted as a covariate in the single-trait GWAS model and the multi-trait test statistic was re-calculated. The conditional analysis revealed that the associated region on BTA6 consisted of two distinct closely located QTL [See Additional file 2: Figure S2]. The QTL located at 88,723,742 bp was associated with udder depth, central ligament and fore udder length, whereas the second QTL located at 90,366,765 bp was associated with teat thickness (Table 2 and [See Additional file 2: Figure S2]). No significant associations were detected for any of the other QTL when the association analysis was conditioned on the respective top SNPs.

**Fig. 3:**
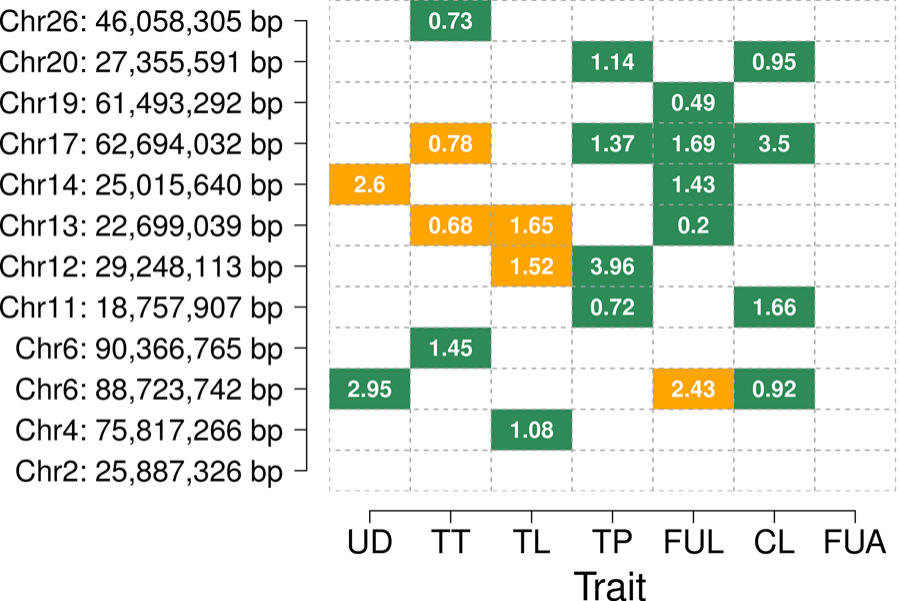
Effects of 12 QTL on seven udder conformation traits. *Green* and *orange* colors represent positive and negative phenotype × genotype associations, respectively, for 12 QTL in seven single-trait association studies. The values in the *rectangles* represent the proportion of DYD variance explained for each QTL. The abbreviations of the traits are in Table 1. Only associations with a P less than 2.97 × 10^−9^ (5 % Bonferroni-corrected significance threshold for 16,816,809 tests) are shown

Seven QTL were associated with multiple morphological features of the mammary gland (Table 2 and Fig. 3). The QTL on BTA17 was associated with four phenotypes. Two and four QTL were associated with three and two udder conformation traits, respectively.

### Genes located in the 12 QTL regions detected in the meta-analysis

To detect positional and functional candidate genes that could be involved in mammary gland morphology, we examined the gene content of 12 QTL regions that were identified in the meta-analysis [See Additional file 3: Figure S3]. However, no annotated genes were present within 300-kb flanking regions of the top association signal on BTA4, 13, 19 and 20, which precluded the identification of positional candidate genes.

Seventy-seven variants with a P_META_ value less than 1 × 10^−8^ were located within a 100-kb intergenic segment on BTA2 [See Additional file 3: Figure S3a]. The top association signal (P_META_ = 6.11 × 10^−10^) was detected for a variant (rs718110021 at 25,887,326 bp) that was located 86 kb downstream of the stop codon of the *MYO3B* gene (*myosin IIIB*) and 111 kb upstream of the translation start site of the *SP5* gene (*Sp5 transcription factor*).

A QTL region on BTA6 included 1199 variants with a P_META_ value less than 1 × 10^−14^ that were located within a 450-kb segment (between 88,613,408 and 89,062,806 bp) [See Additional file 3: Figure S3c]. Two annotated genes reside within this interval: *GC* (*group specific component*) and *NPFFR2* (*neuropeptide FF receptor 2*). The top variant (rs436532576 at 88,723,742 bp; P_META_ = 7.44 × 10^−36^) was located in the first intron of *GC*. The most significantly associated coding variant (rs110326785 or *NPFFR2*: pE389 K; P_META_ = 1.86 × 10^−16^) was considerably less significantly associated than the non-coding top variant. A second QTL on BTA6 was located 6 kb downstream of the stop codon of *RASSF6* (*Ras association (RalGDS/AF*-*6) domain family member 6*) [See Additional file 3: Figure S3d].

Eighty-one variants located within a 1.5-Mb interval on BTA11 (between 18,546,414 and 20,048,201 bp) had P_META_ values less than 3.1 × 10^−10^ [See Additional file 3: Figure S3e]. This segment encompasses 12 annotated transcripts/genes. The top variant (rs384497140 at 18,757,907 bp; P_META_ = 7.47 × 10^−13^) was located 77 kb upstream of the translation start site of *CRIM1* (*cysteine rich transmembrane BMP regulator 1*). Two highly significantly associated coding variants (P_META_ < 1.58× 10^−10^) in *CRIM1* (rs110451648, p.R540 K) and *PRKD3* (rs380476420, p.R861H) were in high LD (r^2^ > 0.88) with the top non-coding variant.

One hundred and twelve variants with a P_META_ value less than 1 × 10^−30^ were located in a 4.59-Mb interval (between 25,693,051 and 30,288,956 bp) on BTA12 [See Additional file 3: Figure S3f]. The top association signal (P_META_ = 1.58 × 10^−42^) was found for an intronic variant (rs381918313 at 29,248,113 bp) in *RXFP2* (*relaxin/insulin*-*like family peptide receptor 2*). None of the highly significantly associated variants were located in the coding region of an annotated gene.

The QTL on BTA14 was in a genomic region that controls growth-related traits in cattle [25, 26] and other species [27]. The top variant (rs109815800 at 25,015,640 bp, P_META_ = 6.23 × 10^−26^) was located 6 kb upstream of the translation start site of *PLAG1* (*pleiomorphic adenoma gene 1*) [See Additional file 3: Figure S3h]. We performed an association analysis with DYD for height at the sacral bone to test if the QTL for mammary gland morphology was also associated with stature. The association analysis revealed that rs109815800 was also the most significantly associated variant for height at the sacral bone (P = 1.07 × 10^−52^) [See Additional file 4: Figure S4]. The allele that increases body height was associated with an increased udder base.

Eighty sequence variants with a P_META_ value less than 1.87 × 10^−25^ were located within a 171-kb intergenic segment (between 62,667,848 and 62,838,591 bp) on BTA17 [See Additional file 3: Figure S3i]. The top variant (rs109134926 at 62,694,032 bp; P_META_ = 2.23 × 10^−57^) was located 193 kb upstream of the translation start site of *RBM19* (*RNA binding motif protein 19*), and 113 kb downstream of the stop codon of *TBX5* (*T*-*box 5 transcription factor*).

A QTL on BTA26 was associated with teat thickness. Thirty-nine variants with a P_META_ value less than 2.0 × 10^−13^ were located in the third intron of *ADAM12* (*ADAM metallopeptidase domain 12*) [See Additional file 3: Figure S3l]. The top variant (rs42442597 at P_META_ = 1.29 × 10^−14^) was located at 46,058,305 bp. No coding variants were significantly associated with mammary gland morphology.

## Discussion

Sequence variants were extrapolated into 10,363 animals using a two-step genotype imputation approach. Initially, the animals with medium-density genotypes were imputed to higher density using 3332 reference animals that had been genotyped with a high-density genotyping array. In the second step, the (partly imputed) high-density genotypes were imputed to sequence level using sequence variants of 157 key ancestors of the Fleckvieh population as reference animals. According to Brøndum et al. [28], using a multi-breed reference panel may improve imputation accuracy when the number of reference animals is small. However, the imputation pipeline that we applied was expected to yield high imputation accuracy by using 157 key ancestors as reference animals [5]. Low-frequency variants occurred more often among the imputed than the array-derived variants. This agrees with previous findings in cattle [6] and other species [29]. Since the imputation of rare variants is error prone [3, 5, 28], we retained only variants with a MAF higher than 0.5 % for the association studies. Moreover, to take imputation uncertainty into account [30], we used the expected allele dosages instead of the most likely genotypes as explanatory variables in the GWAS. Thus, our association analyses should not be flawed due to inaccurately imputed sequence variants.

To eliminate false positive association signals due to population stratification, the genomic relationship based on genome-wide SNP data was considered in the seven separate association studies. The low inflation factors obtained (1.04 to 1.08) show that this corrective measure was successful. Combining the results of the seven separate association studies by calculating an approximate multi-trait test statistic enabled us to reveal 12 QTL for mammary gland morphology. Among these, we detected a QTL on BTA2 that was not found in the single-trait studies, which showed the enhanced capacity of multi-trait approaches for detecting QTL, particularly when the phenotypes are correlated [9, 10, 31]. The QTL on BTA2 was associated with four correlated traits, although not on a genome-wide scale. Our finding that QTL, which cannot be detected at a genome-wide significance level in single-trait GWAS, can be uncovered in a multi-trait approach corroborates the findings of O’Reilly et al. [31]. The joint association analysis of multiple phenotypes might be a more powerful approach to detect QTL that underlie correlated traits than the multi-trait test statistic applied in our study [9]. However, phenotypic records were incomplete for some animals in this study, which precluded multivariate association testing without relying on algorithms to estimate missing phenotypes. Four QTL were detected in the single-trait association studies but were not formally identified in the multi-trait meta-analysis. However, the corresponding P_META_ values were only slightly above the Bonferroni-corrected genome-wide significance threshold. Adjusting for multiple testing using Bonferroni-correction assumes that the individual tests are independent from each other. Due to the small effective population size and high LD, this assumption does not hold for genome-wide association studies in livestock populations. The Bonferroni-correction method is prone to over-correction, particularly in association studies that involve millions of sequence variants [32, 33], which results in reduced power.

Previously, association studies for udder conformation traits were carried out in several cattle breeds using dense marker maps. Flury et al. [34] identified two QTL for udder length and teat diameter in the Brown Swiss cattle breed on BTA6 located at 88.92 and 90.37 Mb, respectively. We also identified two QTL on BTA6 at 88.72 and 90.37 Mb, which confirms the crucial role of both regions for mammary gland morphology in cattle. In our study, the QTL on BTA6 were associated with teat thickness and fore udder length, central ligament and udder depth. Hiendleder et al. [35] identified a QTL for udder conformation traits in the Holstein breed on BTA6 at 88 cM, which most likely corresponds to the highly significantly associated region(s) identified in our study. Another QTL that affects mammary gland morphology in cattle was reported on BTA17, close to *TBX3*, *TBX5* and *RBM19* [34, 36]. The corresponding human chromosome segment is involved in ulnar mammary syndrome [37]. We identified a highly significantly associated QTL at that position with the top variant being only 3 kb away from the top association signal reported in the Brown Swiss breed [34], which indicates that udder traits may be under the control of a common variant in both breeds. The position of this QTL suggests that regulatory variants may be involved in shaping the mammary gland. An improved functional annotation of the bovine genome [38] and multi-breed association studies with imputed sequence variants may reveal causal mutations for such QTL.

The most significantly associated variants at 11 QTL were imputed sequence variants, which demonstrates that the mapping resolution is increased with whole-genome sequence data relative to array-derived genotypes. However, the top association signal was found for an array-derived variant (rs109815800) at a QTL on BTA14. Interestingly, rs109815800 is one of the eight candidate causative trait variants that were reported for a QTL that affects bovine stature by modulating the expression of *PLAG1* [25]. In our study, rs109815800 was also the top variant for body height, which suggests that it has pleiotropic effects on stature and udder traits. The allele that increased height was also associated with an increased udder base. Udder depth, i.e. the interspace between the ankle and the udder base, is visually examined during the evaluation of first crop daughters of artificial insemination bulls and can be overestimated in tall animals. Thus, the association between *PLAG1* and udder depth may reflect phenotypic variation in body size rather than true effects on mammary gland morphology. The fact that this association was not found when udder depth was conditioned on body height further supports this assumption [See Additional file 5: Figure S5].

Our association study revealed new candidate genes for mammary gland morphology in cattle, i.e.:

1. *SP5*, that is located near a QTL on BTA2: since *SP5* is a downstream target gene of the Wnt signaling pathway [39], our result provides additional evidence for the crucial role of the Wnt signaling pathway for mammary gland development in cattle [36];
2. *GC* and *NPFFR2* that are within a QTL on BTA6 and are known to affect mastitis susceptibility in cattle [40–42]; in our study, this QTL was associated with udder depth and central ligament and since udder depth, central ligament and mastitis susceptibility are negatively correlated traits [13], this QTL may contribute to the unfavorable genetic correlation between deep udder base and udder health;
3. *CRIM1* that is located close to a QTL on BTA11 and contains an insulin-like growth factor-binding domain [43];
4. *RXFP2* near a QTL on BTA12;
5. *ADAM12* for which a QTL on BTA26 is located in an intronic region and which encodes the ADAM metallopeptidase domain 12 that interacts with insulin-like growth factor-binding proteins [44]; these findings suggest a crucial role of insulin-like growth factors and insulin-like growth factor-binding proteins during mammary gland development [45, 46].

Most of the QTL that we identified in this study reside in non-protein coding regions of the genome, which indicates that mutations in the regulatory regions of genes have a major role in shaping mammary gland morphology in cattle. Pinpointing causal mutations in non-coding elements is notoriously difficult since the annotation of the bovine genome is not always reliable due to assembly errors and the presence of gaps in the reference sequence [22]. Moreover, regulatory elements in the bovine genome are poorly characterized. Thus, we did not attempt to identify candidate causal variants for QTL. However, an improved functional annotation of the bovine genome is expected to facilitate a more precise characterization of regulatory QTL in the future [38].

## Conclusions

Association analyses based on imputed sequence variants allows the characterization of QTL at maximum resolution. Variants that affect multiple correlated traits are most efficiently uncovered by their simultaneous analysis using a multi-trait test statistic. Our study revealed 12 QTL that control different features of the mammary gland morphology in the German Fleckvieh cattle population. The positions of the QTL suggest that mutations in the regulatory elements have a major role in shaping mammary gland morphology in cattle.

## Additional files

10.1186/s12711-016-0190-4 Manhattan plots representing the association between 16,816,809 imputed sequence variants and seven udder conformation traits.
10.1186/s12711-016-0190-4 Detailed view of the QTL on BTA6.
10.1186/s12711-016-0190-4 Gene content within 12 QTL regions.
10.1186/s12711-016-0190-4 A QTL for stature on BTA14.
10.1186/s12711-016-0190-4 Detailed view of a QTL for mammary gland morphology on BTA14.
