# Supplementary figures and images for: A multi-trait meta-analysis with imputed sequence variants reveals twelve QTL for mammary gland morphology in Fleckvieh cattle
Source: Genet Sel Evol. 2016 Feb 16;48:14. doi: 10.1186/s12711-016-0190-4 (PMC4756527; doi:10.1186/s12711-016-0190-4)

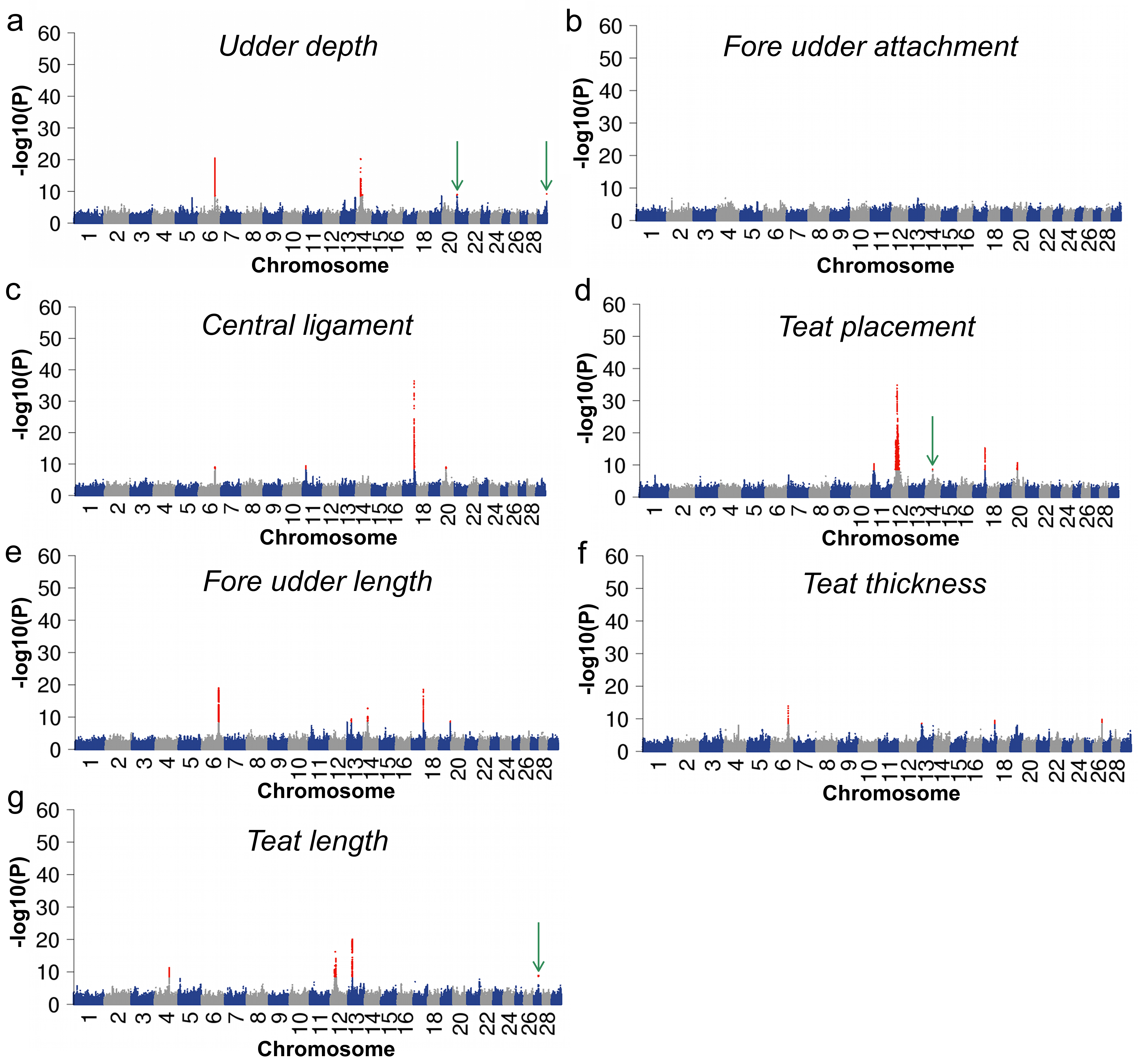

Supplement: Supplementary file 1 — 10.1186/s12711-016-0190-4 Manhattan plots representing the association between 16,816,809 imputed sequence variants and seven udder conformation traits. [file 12711_2016_190_MOESM1_ESM.tif]

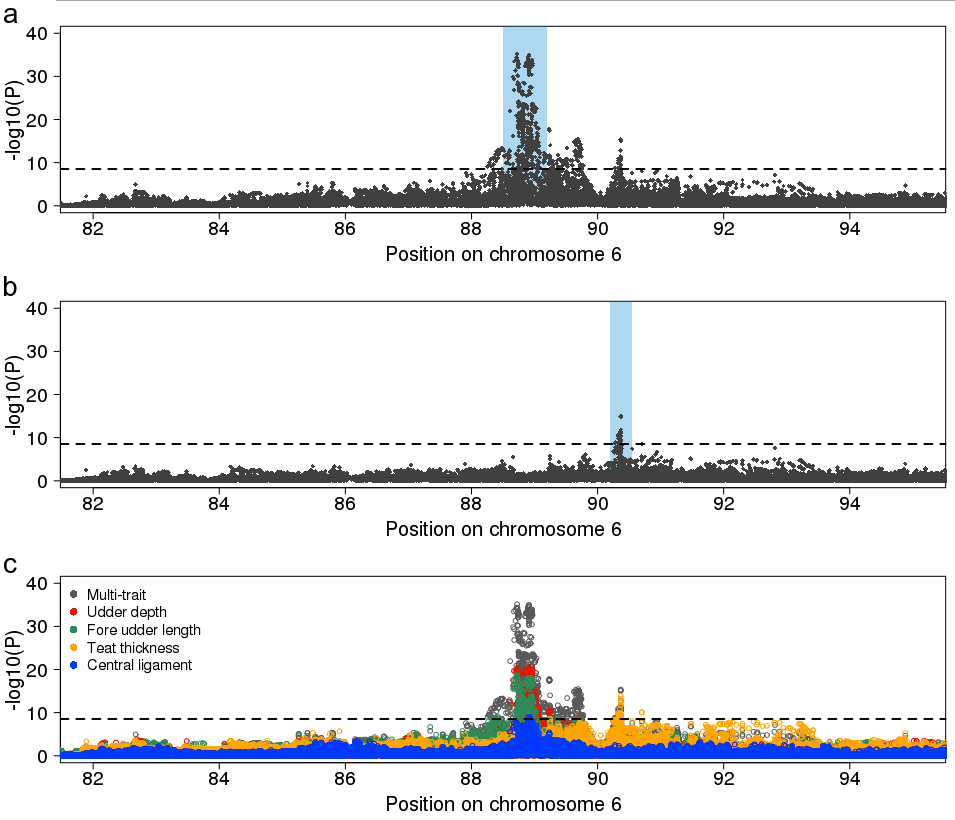

Supplement: Supplementary file 2 — 10.1186/s12711-016-0190-4 Detailed view of the QTL on BTA6. [file 12711_2016_190_MOESM2_ESM.png]

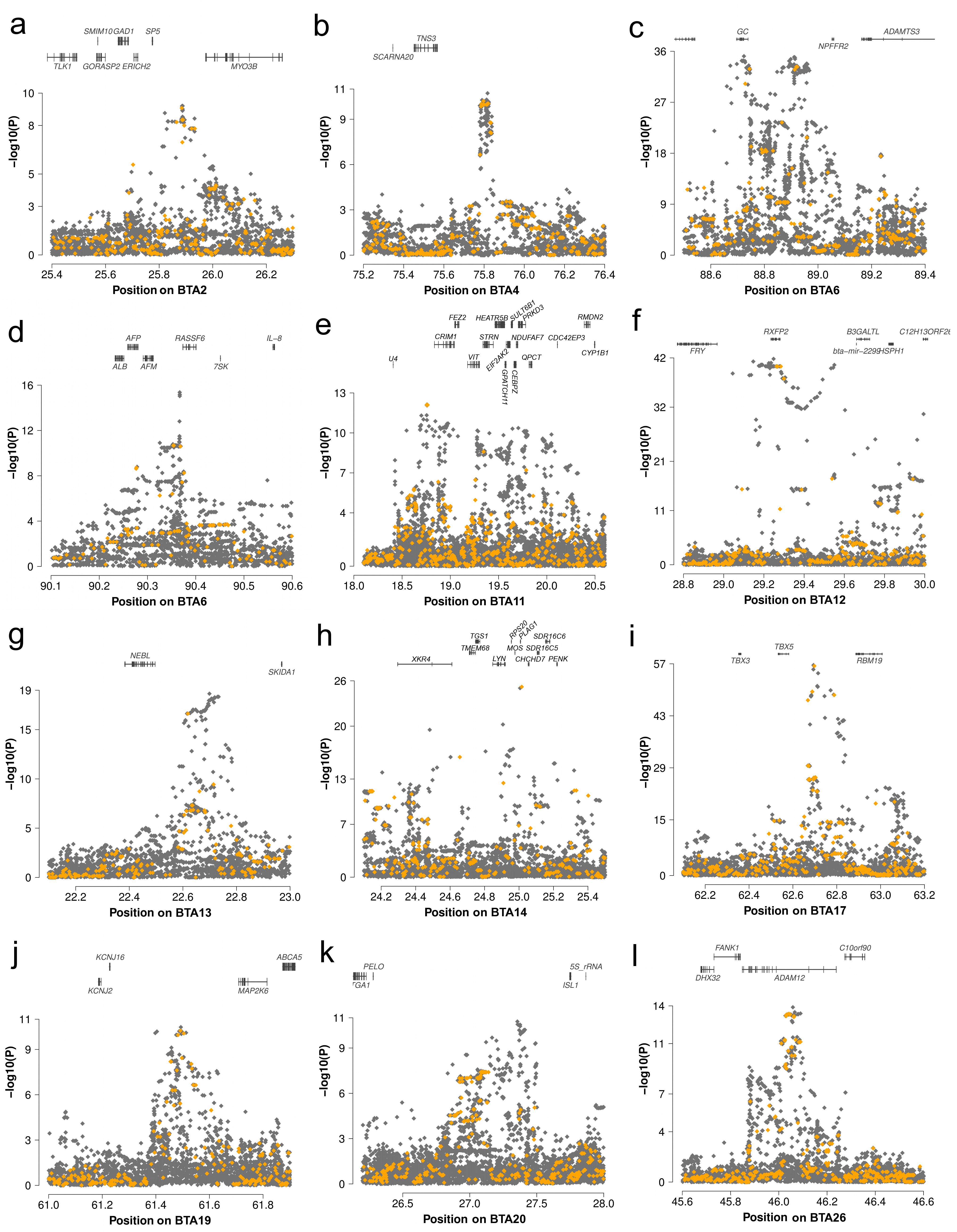

Supplement: Supplementary file 3 — 10.1186/s12711-016-0190-4 Gene content within 12 QTL regions. [file 12711_2016_190_MOESM3_ESM.tif]

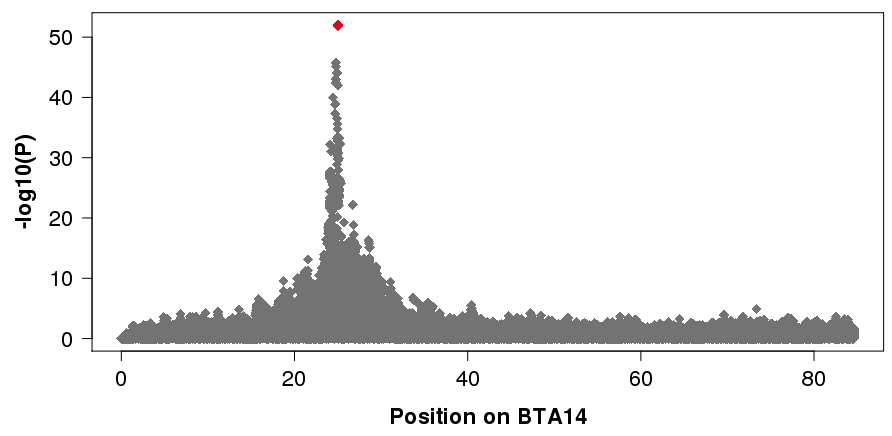

Supplement: Supplementary file 4 — 10.1186/s12711-016-0190-4 A QTL for stature on BTA14. [file 12711_2016_190_MOESM4_ESM.png]

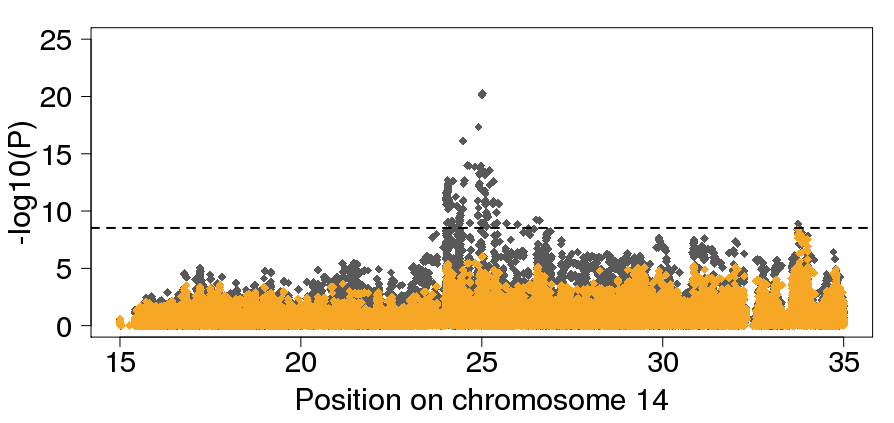

Supplement: Supplementary file 5 — 10.1186/s12711-016-0190-4 Detailed view of a QTL for mammary gland morphology on BTA14. [file 12711_2016_190_MOESM5_ESM.png]
